# Metabolic Perturbations Caused by the Over-Expression of *mcr-1* in *Escherichia coli*

**DOI:** 10.3389/fmicb.2020.588658

**Published:** 2020-10-09

**Authors:** Yi-Yun Liu, Yan Zhu, Hasini Wickremasinghe, Phillip J. Bergen, Jing Lu, Xiao-Qing Zhu, Qiao-Li Zhou, Mohammad Azad, Sue C. Nang, Mei-Ling Han, Tao Lei, Jian Li, Jian-Hua Liu

**Affiliations:** ^1^National Risk Assessment Laboratory for Antimicrobial Resistance of Animal Original Bacteria, Guangdong Provincial Key Laboratory of Veterinary Pharmaceutics Development and Safety Evaluation, Key Laboratory of Zoonosis of Ministry of Agricultural and Rural Affairs, College of Veterinary Medicine, South China Agricultural University, Guangzhou, China; ^2^Biomedicine Discovery Institute and Department of Microbiology, School of Biomedical Sciences, Monash University, Clayton, VIC, Australia; ^3^Guangdong Laboratory for Lingnan Modern Agriculture, Guangzhou, China; ^4^State Key Laboratory of Applied Microbiology Southern China, Guangdong Institute of Microbiology, Guangdong Academy of Sciences, Guangzhou, China

**Keywords:** colistin, *mcr-1*, fitness cost, metabolomics, glycerophospholipids, pentose phosphate pathway, pantothenate and CoA biosynthesis

## Abstract

Rapid dissemination of the plasmid-born polymyxin resistance gene *mcr-1* poses a critical medical challenge. MCR-1 expression is tightly controlled and imposes a fitness cost on the bacteria. We used growth studies and metabolomics to examine growth and metabolic changes within *E. coli* TOP10 at 8 and 24 h in response to different levels of expression of *mcr-1*. Induction of *mcr-1* greatly increased expression at 8 h and markedly reduced bacterial growth; membrane disruption and cell lysis were evident at this time. At 24 h, the expression of *mcr-1* dramatically declined with restored growth and membrane integrity, indicating regulation of *mcr-1* expression in bacteria to maintain membrane homeostasis. Intermediates of peptide and lipid biosynthesis were the most commonly affected metabolites when *mcr-1* was overexpressed in *E. coli*. Cell wall biosynthesis was dramatically affected with the accumulation of lipids including fatty acids, glycerophospholipids and lysophosphatidylethanolamines, especially at 8 h. In contrast, levels of intermediate metabolites of peptides, amino sugars, carbohydrates and nucleotide metabolism and secondary metabolites significantly decreased. Moreover, the over-expression of *mcr-1* resulted in a prolonged reduction in intermediates associated with pentose phosphate pathway and pantothenate and CoA biosynthesis. These findings indicate that over-expression of *mcr-1* results in global metabolic perturbations that mainly involve disruption to the bacterial membrane, pentose phosphate pathway as well as pantothenate and CoA biosynthesis.

## Introduction

The emergence and spread of multidrug-resistant (MDR) and extensively drug-resistant (XDR) Gram-negative bacteria represents an increasing threat to global public health (McEwen and Collignon, 2018; Lerminiaux and Cameron, 2019). Due to a shortage of effective antimicrobial drugs to combat these “superbugs,” particularly carbapenemase-producing Enterobacteriaceae, use of the once sidelined polymyxin antibiotics (polymyxin B and colistin) has dramatically increased over the last two decades (Falagas and Kasiakou, 2005; Karaiskos and Giamarellou, 2014). The bactericidal activity of the polymyxins begins with their binding to the lipid A component of lipopolysaccharides (LPS) in the Gram-negative bacterial outer membrane (OM) via both electrostatic and hydrophobic interactions, which is followed by membrane disorganization and cell lysis (Velkov et al., 2013; Olaitan et al., 2014). Intrinsic resistance involves sensing the presence of polymyxins in the environment via two-component regulatory systems (TCSs, e.g., PmrAB and PhoPQ). These TCSs upregulate the expression of *pmrC* and/or *arnBCADTEF-ugd* leading to the modification of the phosphate groups of lipid A via the addition of cationic 4-amino-4-deoxy-L-arabinose (L-Ara4N) and/or phosphoethanolamine (pEtN) moieties, thereby attenuating the disorganization of the OM (Falagas and Kasiakou, 2005; Olaitan et al., 2014). Alarmingly, reports of polymyxin resistance are increasing worldwide (Velkov et al., 2013; Karaiskos and Giamarellou, 2014; Olaitan et al., 2014; Bonomo et al., 2018). The mechanisms of polymyxin resistance in these organisms include mutations in *pmrAB* and *phoPQ* and the negative regulator *mgrB* (in *Klebsiella pneumoniae*) that lead to constitutive expression of lipid A modification genes, and mutations in lipid A biosynthesis genes (e.g., *lpxACD*) that cause the loss of LPS and therefore prevent polymyxin binding (Cannatelli et al., 2013; Olaitan et al., 2014).

All the aforementioned resistance mechanisms are chromosomally mediated; however, this paradigm has changed significantly since we reported the first case of plasmid-mediated polymyxin resistance in 2015 (Liu et al., 2016). The mobilized colistin resistance gene, *mcr-1*, has been detected globally in various Gram-negative bacteria collected from food, live-stock, companion animals, rivers, vegetables and humans (Liu and Liu, 2018). Other *mcr* family genes (*mcr-2* to *mcr-10*) that confer polymyxin resistance have also been reported worldwide (Carroll et al., 2019; Nang et al., 2019; Wang et al., 2020). MCR-1 is an inner-membrane-anchored protein consisting of a periplasmic *C*-terminal domain and an *N*-terminal transmembrane domain (Liu et al., 2016). Belonging to the phosphoethanolamine (pEtN) transferase family, MCR-1 catalyzes the transfer of pEtN from phosphatidylethanolamine (PE) to the phosphate groups of lipid A (Liu et al., 2017). Compared to chromosomally mediated high-level polymyxin resistance that typically results in polymyxin minimum inhibitory concentrations (MICs) in the range of 8–256 mg/L, increases in polymyxin MICs mediated by *mcr-1* are more modest (generally 2–8 mg/L) indicating a potential fitness cost owing to *mcr-1* expression (Bontron et al., 2016; Denervaud Tendon et al., 2017); though organism dependent, the MIC breakpoint for polymyxin susceptibility is typically ≤2 mg/L. Indeed, significant damages to the cell envelope, slow growth rates and cell lysis have been observed following induced expression of *mcr-1* in *E. coli* (Yang et al., 2017; Tietgen et al., 2018); however, the underlying mechanisms of such defects caused by *mcr-1* expression remain unclear.

Metabolomics is able to identify critical bacterial metabolic changes in response to antibiotic treatments at the network level (Jozefczuk et al., 2010; Zampieri et al., 2017). Using metabolomics, Li H. et al. (2019) found that *mcr-1* primarily affected metabolic pathways involved in glycerophospholipid metabolism and biosynthesis of LPS and phosphatidylethanolamine (PE). It is likely that clinical isolates of *E. coli* containing *mcr-1* have already undergone long-term adaptation via antibiotic selection and that cellular metabolism has been fine-tuned to cope with moderate *mcr-1* expression. In the present study, we employed metabolomics to investigate the dynamic metabolic changes occurring in response to different levels of *mcr-1* expression in *E. coli* and elucidate the mechanisms of metabolomic adaptation. This is the first comprehensive study of metabolic changes in response to *mcr-1* expression in *E. coli*. Our findings will assist in the prevention of *mcr-1* dissemination and facilitate the optimization of polymyxin therapy.

## Materials and Methods

### Strains and Plasmids

*E. coli* TOP10 was used as the recipient strain of different plasmids (Supplementary Table S1). The expression vector pBAD-hisA (Thermo Fisher Scientific, United States) was used to construct recombinant plasmids. pBAD-hisA contains a promoter for the *araBAD* (arabinose) operon that controls the expression of genes, which can be regulated with L-Arabinose (Guzman et al., 1995). *mcr-1* was cloned from pHNSHP45 (GenBank: KP347127.1) using primer pair (*mcr-1*-NS) to pBAD-hisA, yielding pBAD-*mcr-1* (Supplementary Tables S1, S2). The recombinant plasmid was confirmed by Sanger sequencing and used to transform *E. coli* TOP10 by electroporation (Liu et al., 2017). *E. coli* TOP10 were transformed with empty vector pBAD as controls. Cells were plated on lysogeny broth (LB) agar plates containing ampicillin at 100 mg/L. The introduction of pBAD, pBAD-*mcr-1* was confirmed by PCR using primers *mcr-1*-NS and pBAD-NS (Supplementary Table S2), respectively. Minimum inhibitory concentrations (MICs) of colistin (sulfate; Beta Pharma, Shanghai, China) were determined by broth microdilution in cation-adjusted Mueller-Hinton broth (CAMHB; Oxoid, VIC, Australia) containing 25 mg/L Ca^2+^ and 12.5 mg/L Mg^2+^ supplemented with different concentrations of L-arabinose (0, 0.02, 0.2, and 2%, w/v). *E. coli* ATCC 25922 was used as the reference strain for MIC measurement (Clinical and Laboratory Standards Institute, 2017).

### Quantitative Real-Time PCR (qRT-PCR)

Overnight cultures of strain *E. coli* TOP10 carrying pBAD-*mcr-1* were diluted (1:100) in 5 mL of fresh CAMHB (containing 25 mg/L Ca^2+^ and 12.5 mg/L Mg^2+^) containing different concentrations of L-arabinose (0, 0.02, 0.2, and 2%, w/v) and incubated with shaking (180 rpm) at 37°C for 24 h. Bacterial samples (*n* = 3) were collected at 0, 4, 8, and 24 h for viable cell counting and at 4 and 8 h for RNA extraction. Trizol (Invitrogen, United States) was employed to extract RNA. RNase-free DNase (QIAGEN, Germany) was added to remove DNA residue and a secondary phenol:chloroform purification by 5PRIME Phase Lock Gel tubes (Quanta Biosciences, United States) was then performed (Gulliver et al., 2018). Complementary DNA was synthesized using the prepared RNA and an AffinityScript qPCR cDNA synthesis kit (Agilent Technologies, United States). qPCR was conducted using QuantiNova SYBR Green RT-PCR Kit (QIAGEN, Germany) and an Agilent AriaMx real-time PCR system (Agilent Stratagene, United States); *rpoB* was used as a reference gene. The primer sequences are shown in Supplementary Table S2. Relative expression results were normalized by the ΔΔCT analysis method using mean CT value. Data acquisition and analysis were performed using a non-parametric Mann-Whitney *U* test or one-way ANOVA according to Dunn’s multiple comparison test.

### Cellular Morphology Analysis

Overnight cultures of *E. coli* TOP10 carrying pBAD and pBAD-*mcr-1* were diluted (1:100) in 5 mL fresh CAMHB (25 mg/L Ca^2+^ and 12.5 mg/L Mg^2+^) without or with L-arabinose (0.2%) and incubated with shaking (180 rpm) at 37°C for 24 h. Bacterial samples were collected at 8 and 24 h for observation of cell membrane integrity by transmission electron microscope (TEM). In brief, samples were collected and washed twice with cold 0.9% sterile saline then centrifuged for 10 min at 3,220 g and 4°C. The supernatant was then discarded leaving the cell pellet which was subsequently re-suspended with 2.5% glutaraldehyde fixative, fixed at 4°C for 2 h, washed for 4 × 30 min in phosphate buffer saline (PBS) and then fixed in osmic acid followed by dehydration using graded ethanol (30, 45, 60, 75, 85, and 95% for 15 min each, 100% for 2 × 20 min). The precipitates were twice washed with acetone for 20 min, once with acetone:resin (3:1) for 12 h, once with acetone:resin (1:1) for 4 h and once with resin for 1 h before embedding in Epon812 resin. The samples were sectioned into slices of approximately 50 nm width with an ultramicrotome before staining with lead citrate and imaged using TEM at 80 kV.

### Metabolite Extraction

Cellular metabolites were extracted as described previously with minor modifications (Han et al., 2018). In brief, 100 mL bacterial cultures (*n* = 5) with and without 0.2% L-arabinose induction were harvested at 8 and 24 h and immediately transferred to an ethanol:dry ice bath for 30-s quenching. The samples were then normalized to OD_600_ 0.50 ± 0.02 (600 nm optical density). Cells were pelleted from 20 mL of the normalized sample by centrifugation (3,220 × g, 4°C, 10 min), washed twice with cold 0.9% sterile saline, and resuspended in 0.5 mL chloroform:methanol:water (1:3:1, v/v) containing 1 μM generic internal standards (CHAPS, CAPS, PIPES, and TRIS). Three freeze-thaw cycles were conducted in liquid nitrogen to lyse the cells and completely release cellular metabolites. The solvents were then centrifuged for 10 min at 3,220 g and 4°C after which 0.3 mL of the supernatant was subject to a secondary centrifugation at 14,000 g for 15 min at 4°C to obtain 0.2 mL particle-free supernatants for LC-MS analysis.

### LC-MS Analysis

The particle-free supernatant samples were analyzed on a Q-Exactive Orbitrap mass spectrometer (MS, Thermo Fisher Scientific), in tandem with a DionexU3000 high-performance liquid chromatograph (HPLC, Thermo Fisher Scientific) with a ZIC-pHILIC column (5 μm, polymeric, 150 × 4.6 mm; SeQuant, Merck). The MS system was operated in both positive and negative electrospray ionization (ESI) ion mode with a resolution at 35,000 and a detection range of 85 to 1,275 *m/z*. Samples were eluted by a multi-step gradient system at a flow rate of 0.3 mL/min beginning with 20% mobile phase A (20 mM ammonium carbonate) and 80% mobile phase B (acetonitrile) to 50% mobile phase A and 50% mobile phase B over 15 min, followed 5% mobile phase B for 3 min. Re-equilibration with 20% mobile phase A and 80% mobile phase B for 8 min was then performed. All sample with internal standards were separately analyzed in a single LC-MS batch to minimize operational variations. The pooled biological quality control (PBQC) samples (aliquot of 10 μL of each sample) were analyzed throughout the batch to assess chromatographic peaks, signal reproducibility and analyte stability. Mixtures of authentic standards containing about 350 metabolites were analyzed within the same batch for the identification of metabolites.

### Data Processing and Statistics Analysis

Raw data were processed by mzMatch and IDEOM^1^ to determine the intensity of metabolites (Creek et al., 2012). The intensities were normalized by the median, log transformed, auto scaled, then analyzed by principal-component analysis (PCA) and one-way analysis of variance (ANOVA, Tukey’s honestly significant difference [HSD] *post-hoc* test) using MetaboAnalyst 4.0 (Chong et al., 2018). Differentially abundant metabolites (false discovery rate [FDR] adjusted *P* < 0.05, fold change [FC] ≥ 2) were used for pathway analysis using Kyoto Encyclopedia of Genes and Genomes (KEGG) (Kanehisa and Goto, 2000) and BioCyc (Caspi et al., 2018) databases.

## Results

### Over-Expression of *mcr-1* Reduced Bacterial Growth

Colistin MICs in the absence or presence of L-arabinose are shown in Supplementary Table S1. The MICs of the vector control strain *E. coli* pBAD were 0.5 mg/L in the absence of L-arabinose and in the presence of all three L-arabinose concentrations tested (0.02, 0.2, and 2%). While the MIC of *E. coli* TOP10 + pBAD-*mcr-1* similarly remained 0.5 mg/L with 0 and 0.02% L-arabinose, the MIC increased 4-fold to 2 mg/L in the presence of 0.2% L-arabinose but dropped to 1 mg/L in the presence of 2% L-arabinose.

**FIGURE 1:**
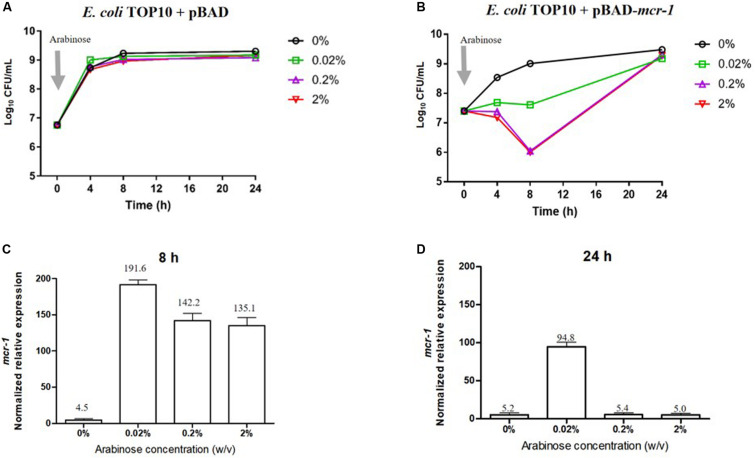
Impact of *mcr-1* expression on bacterial growth. Bacterial growth curves of *E. coli* TOP10 carrying **(A)** the empty vector pBAD and **(B)** pBAD-*mcr-1* under different levels of Arabinose induction. The expression levels of *mcr-1* were measured by qRT-PCR. The normalized relative expression (mean ± SD, *n* = 3) of *mcr-1* in *E. coli* TOP10 + pBAD-*mcr-1* under different levels of Arabinose induction at **(C)** 8 h and **(D)** 24 h.

Bacterial growth and the level of expression of *mcr-1* in the absence and presence of L-arabinose are shown in Figure 1. Growth of *E. coli* pBAD (containing the empty vector) was completely unaffected by L-arabinose (Figure 1A). In *E. coli* TOP10 + pBAD-*mcr-1* strains, growth in the absence of L-arabinose approximated that of *E. coli* pBAD (Figures 1A,B). However, unlike the vector control strain, the growth of *E. coli* TOP10 + pBAD-*mcr-1* was markedly decreased at 4 and 8 h by 0.02% and, especially, 0.2 and 2% L-arabinose (Figure 1B). By 24 h the growth of *E. coli* TOP10 + pBAD-*mcr-1* had recovered to that observed in the absence of L-arabinose. qPCR results clearly demonstrated *mcr-1* was significantly over-expressed at 8 h following L-arabinose induction at all concentrations (0.02–2%, Figure 1B); however, over-expression was retained at 24 h only in the presence of 0.02% L-arabinose (Figure 1D). Together, these results suggest that *mcr-1* over-expression may cause a significant fitness cost during bacterial exponential growth, and that the bacterial cells may undergo metabolic changes to overcome such a burden and achieve a similar level of viability after 24 h to that of cells which did not express *mcr-1*.

### Over-Expression of *mcr-1* Affected Cell Morphology

**FIGURE 2:**
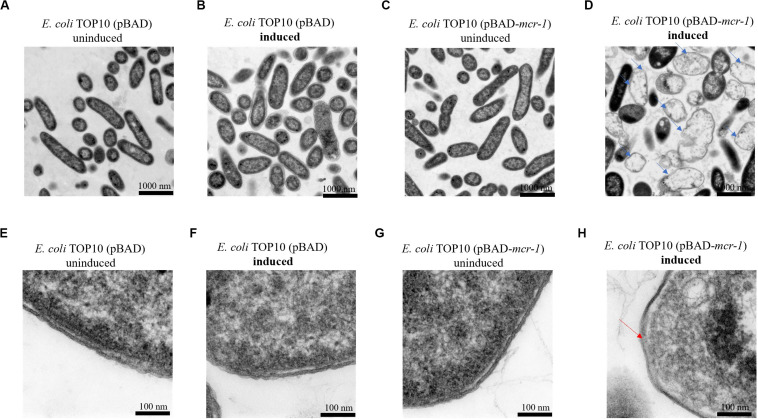
TEM micrographs of *E. coli* at 8 h with or without induction by 0.2% L-arabinose. **(A,B)** show *E. coli* TOP10 + pBAD (the vector control) and **(C,D)** *E. coli* TOP10 + pBAD *mcr-1*. **(E–H)** show partially magnified images of figures **(A–D)**, respectively. All cells in **(A–C)** have an intact membrane and the electron density of cytoplasmic region is homogeneous. Over-expression of *mcr-1* in **(D)** was associated with membrane damage (red arrow) and cell lysis (blue arrows).

TEM micrographs of *E. coli* TOP10 + pBAD and *E. coli* TOP10 + pBAD-*mcr-1* in both the induced (0.2% [w/v] L-arabinose) and uninduced (0% L-arabinose) state are shown in Figure 2 (after 8 h induction) and S1 (after 24 h induction). For *E. coli* TOP10 + pBAD (vector control), at both 8 and 24 h the cell membrane remained intact and the cytoplasm remained compact and homogenous irrespective of the induction by L-arabinose. Similarly, no changes in cellular architecture were observed with uninduced *E. coli* TOP10 + pBAD-*mcr-1* at either time. However, changes in cell morphology of *E. coli* TOP10 + pBAD-*mcr-1* were observed at 8 h following the induction of *mcr-1* by L-arabinose (Figures 2D,H). With these cells, the structural integrity of the membrane was altered as evidenced by the change in a lack of demarcation between the outer and inner membranes (Figure 2H). Cytoplasmic changes including granular degeneration (Figure 2H) and vacuolation (Figure 2D) were also observed, and most cells were lysed. At 24 h, cells that survived this initial perturbation showed normal cellular architecture indistinguishable from uninduced cells (Supplementary Figure S1).

### Global Metabolic Variations in Response to *mcr-1* Over-Expression in *E. coli*

**FIGURE 3:**
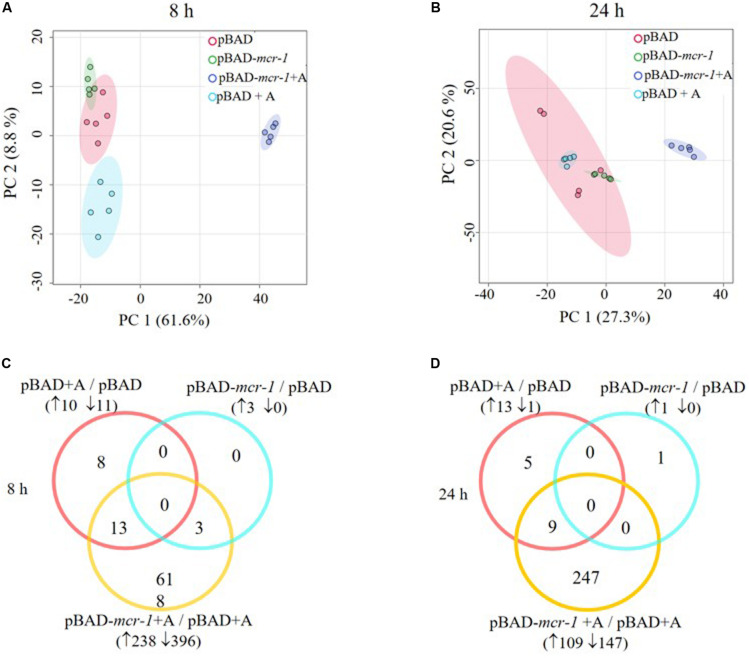
Multivariate and univariate analyses of global metabolic changes. PCA score plots of the first two principle components for metabolite levels from samples (*E. coli* TOP10 carrying different recombinant plasmids) with or without 0.2% Arabinose induction at **(A)** 8 h and **(B)** 24 h. Each dataset represents a total of 30 samples of 5 biological replicates of each condition. Empty vector pBAD without induction = Red (pBAD), with induction = Blue (pBAD + A); pBAD-*mcr-1* without induction = Purple (pBADm_0), with induction = Yellow (pBADm_A). Venn diagrams display the number of metabolites significantly affected by pBAD-*mcr-1*, over-expression of pBAD, or over-expression of pBAD-*mcr-1* at **(C)** 8 h and **(D)** 24 h, relative to the control group. Significant metabolites were selected with FDR < 0.05, *p* < 0.05 and fold change ≥ 2 (one-way ANOVA).

LC-MS based metabolomics was conducted to identify metabolic changes in *E. coli* TOP10 + pBAD and TOP10 + pBAD-*mcr-1* in the absence and presence of 0.2% L-arabinose induction at 8 h and 24 h. A total of 1,101 unique metabolites were putatively identified (Supplementary Dataset S1). The median relative standard deviation (RSD) was 16.5% for PBQC samples, and 20.0–37.0% for sample groups, indicating a well-control technical variation (Supplementary Table S3) (Kirwan et al., 2014). PCA plots revealed that significant metabolic changes occurred in the over-expression strains (TOP10 + pBAD-*mcr-1*) following induction at 8 and 24 h (Figures 3A,B). Further, the significantly changed metabolites (FDR < 0.05, *p* < 0.05, and fold change ≥ 2, ANOVA) were sorted out for further analysis. In detail, in the absence of L-arabinose, only 3 and 1 changed metabolites were induced by pBAD-*mcr-1* in *E. coli* TOP10 at 8 and 24 h (Figures 3C,D), relative to pBAD. In the presence of L-arabinose, compared to pBAD, overexpression of *mcr-1* resulted in 634/256 differentially abundant metabolite in *E. coli* (TOP10 + pBAD-*mcr-1*) at 8/24 h (Figures 3C,D), indicating that over-expression of *mcr-1* caused dramatic perturbations of metabolites in *E. coli*. To eliminate these observations being singularly due to L-arabinose induction, comparative analysis of *E. coli* TOP10 + pBAD to remove changes caused by L-arabinose induction.

### Over-Expression of *mcr-1* Mainly Caused Changes of Bacterial Lipid and Peptide Metabolism

**FIGURE 4:**
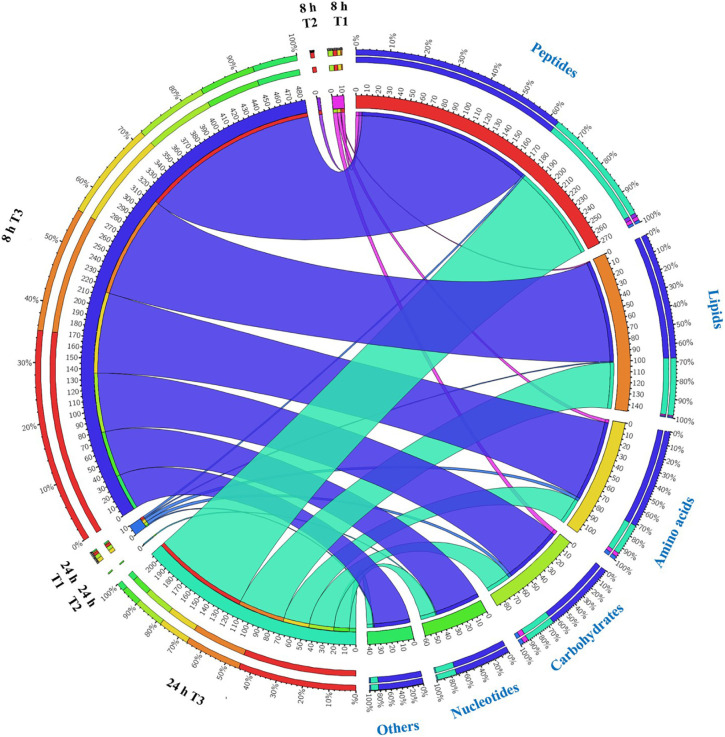
Metabolome response of *E. coil* at 8 h and 24 h. Bipartite graph exhibits correlations between the number of significant metabolites (left side) and significantly affected metabolites (FDR < 0.05, *p* < 0.05 and fold change ≥ 2) (right side, the quantity and proportion). The number of metabolites significantly affected by over-expression of pBAD (T1 = pBAD + Arabinose vs. pBAD), pBAD-*mcr-1* (T2 = pBAD-*mcr-1* vs. pBAD), or over-expression of pBAD-*mcr-1* (T3 = pBAD-*mcr-1* + Arabinose vs. pBAD + Arabinose).

**FIGURE 5:**
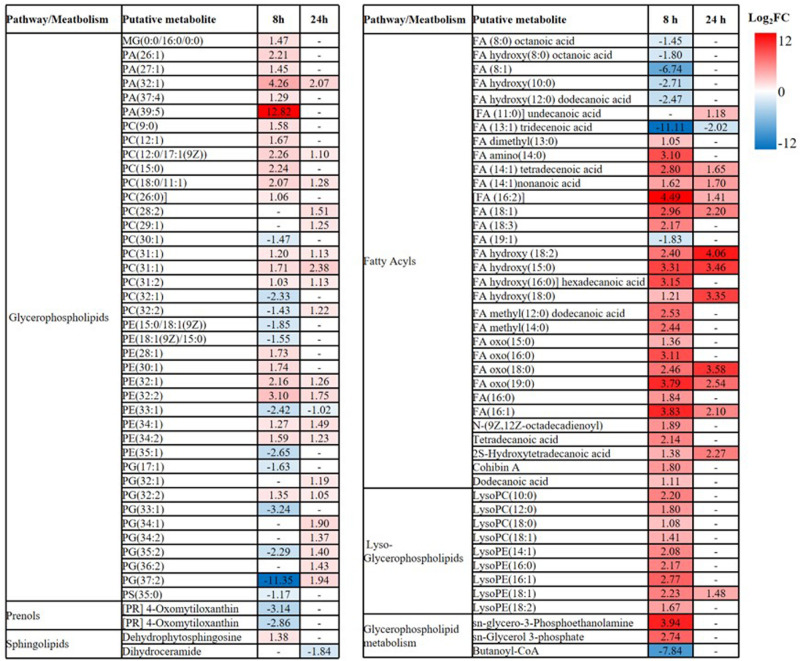
Perturbations of lipids and related metabolites. Lipid names are putatively assigned based on accurate mass. The values and whether metabolite level decreased (blue) or increased (red) are shown. The heatmap is shown to the right of the table with the log_2_ fold change (FC) values. FDR < 0.05, *p* < 0.05 and fold change ≥ 2.

Overall, intermediates of lipids and peptides were the most affected metabolites in the over-expression of *mcr-1* strain (*E. coli* TOP10 + pBAD-*mcr-1*) at 8 and 24 h following L-arabinose induction compared to *E. coli* TOP10 + pBAD (Figure 4). In particular, at 8 h, the accumulation of the medium-chain fatty acids (FA tetradecenoic acid, tridecenoic acid, nonanoic acid, hexadecanoic acid, and dodecanoic acid) was observed (Figure 5). In contrast, the shorter-chain fatty acids [FA (8:0), FA hydroxy (8:0), FA hydroxyl (10:0 and 12:0), and FA (13:1) tridecenoic acid] were depleted at 8 h. Additionally, the level of many glycerophospholipids (GPLs) were significantly increased, including glycerophosphates [PA (26:1, 27:1, 32:1, 37:4, and 39:5)], phosphatidylcholine [PC (9:0, 12:1, 12:0/17:1, 15:0, 18:0/11:1, 26:0)], phosphatidylethanolamine [PE (28:1, 30:1, 32:1, 32:2, and 34:1 and 34:2)], phosphatidylglycerol [PG (32:2)], phosphatidylserine [PS (35:0)], lysophosphatidylcholine [LysoPC (10:1, 12:0, 18:0, and 18:1)], lysophosphatidylethanolamines [LysoPE (14:1, 16:0, 16:1, 18:1, and 18:2)], *sn*-glycero-3-phosphoethanolamine and *sn*-glycerol 3-phosphate (Figure 5). By comparison, other metabolite intermediates for GPL metabolism [e.g., Butanoyl-CoA, PC (30:1), PE (35:1), and PG (17:1, 33:1, 35:0, 35:2, and 37:2] and prenols metabolism were dramatically decreased. At 24 h, over-expression of *mcr-1* mainly involved enrichment of FAs and GPLs, predominantly GLPs [long-chain PC, PE, and PG] and medium-chain fatty acids. Obviously, the over-expression of proteins MCR-1 induced a greater metabolic response in the level of lipid metabolism at 8 h compared to 24 h (Figure 4).

Substantial metabolic variations in the amounts of short-chain peptides were consistently detected (Figure 4). The over-expression of proteins MCR-1 caused greater metabolic changes in peptide levels at 8 h compared to at 24 h. Besides, a number of other metabolic pathways were also affected by MCR-1 over-expression at 8 h yet had returned close to control values by 24 h (Supplementary Dataset S1). For example, at 8 h, a decrease in the levels of xanthosine (*FC* = 159) indicated a reduction in nucleotide metabolism, whereas reductions in secondary metabolites such as *O*-beta-D-Glucopyranosyl-cis-zeatin (*FC* = 219) indicated developmental or morphological changes. By 24 h, the levels of these metabolites had increased and approximated to those of the vector control (Supplementary Dataset S1).

### Over-Expression of *mcr-1* Induced Metabolic Disruptions in Pentose Phosphate Pathway and Pantothenate and CoA Biosynthesis

**FIGURE 6:**
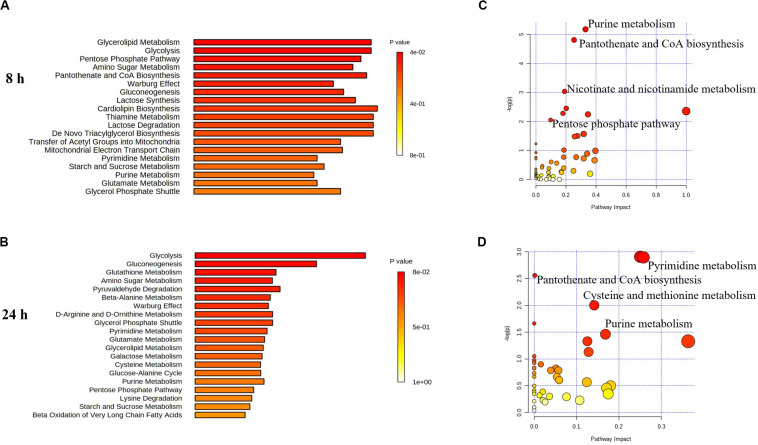
Perturbations of lipids and related metabolites. Enrichment analysis **(A,B)** and pathway analysis of significantly altered metabolites **(C,D)** in *E. coli* TOP10 with over-expression of mcr-1 at 8 and 24 h, respectively. FDR < 0.05, *p* < 0.05 and fold change ≥ 2, one-way ANOVA. The pathway enrichment analysis was based on KEGG Pathway with reference to *E. coli* K-12. The figures were produced by MetaboAnalyst 4.0 (https://www.metaboanalyst.ca/).

**FIGURE 7:**
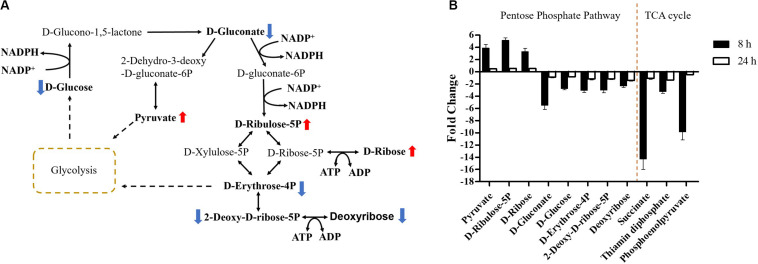
Central metabolic changes in pentose phosphate pathway. **(A)** In the pathway flow charts (adapted from KEGG Pathway with reference to *E. coli* K-12), red arrows and blue arrows indicate that the metabolites were significantly increased or decreased, respectively. **(B)** Metabolites associated with this pathway and TCA cycles at 8 and 24 h. FDR < 0.05, *p* < 0.05 and fold change ≥ 2.

Carbohydrate metabolism was significantly decreased by over-expression of *mcr-1*, with major changes observed for the metabolites of the pentose phosphate pathway (PPP) at 8 h (Figures 4, 6A). A number of key metabolites (e.g., D-gluconate, D-glucose, D-erythrose-4P, 2-deoxy-D-ribose-5P, and deoxyribose) were significantly decreased, whereas the increased relative abundance of three metabolites (e.g., pyruvate, D-ribulose-5P, and D-ribose) were detected (Figures 7A,B). Moreover, the levels of succinate, thiamin diphosphate and phosphoenolpyruvate related to citrate cycle (TCA cycle) were also significantly decreased in response to over-expression of *mcr-1* at 8 h (Figure 7B). By 24 h, the levels of these metabolites had gotten back to those of the control (Supplementary Dataset S1).

**FIGURE 8:**
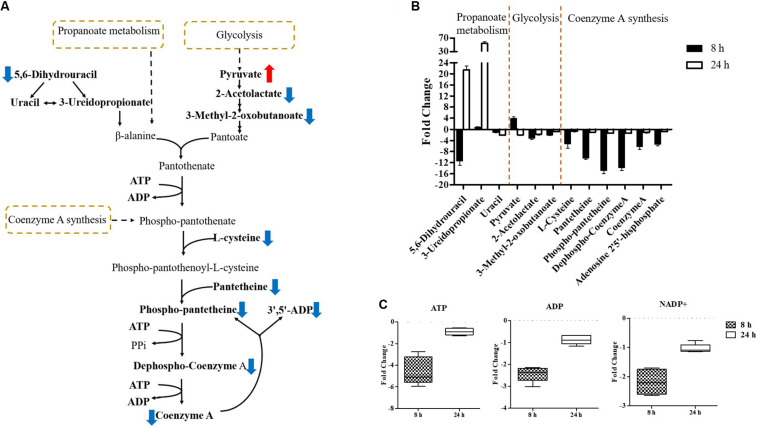
Central metabolic changes in pantothenate and CoA biosynthesis. **(A)** In the pathway flow charts (adapted from KEGG Pathway with reference to *E. coli* K-12), red arrows and blue arrows indicate that the metabolites were significantly increased or decreased, respectively. **(B)** Metabolites associated with this pathway and **(C)** decreased levels of key energy-associated metabolites in *E. coli* TOP10 with over-expression of *mcr-1* at 8 and 24 h. FDR < 0.05, *p* < 0.05 and fold change ≥ 2.

Pathway enrichment analysis revealed that over-expression of *mcr-1* following arabinose induction resulted in significant decreases in pantothenate and CoA biosynthesis (Figure 6). For example, at 8 h, the amounts of intermediate metabolites in pantothenate and CoA biosynthesis (e.g., 5,6-dihydrouracil, 2-acetolactate, 3-Methyl-2-oxobutanoate, L-cysteine, pantetheine, phospho-pantetheine, dephospho-CoA, coenzyme A, 3’,5’-ADP) were dramatically reduced (*FC* > 2), whereas significant accumulation of pyruvate (related to glycolysis) was observed (Figures 8A,B). At 24 h, over-expression only caused four significant changes related to propanoate and glycolysis metabolism, including 5,6-dihydrouracil, pyruvate, uracil, and 3-ureidopropionate (Figures 8A,B), the levels of other metabolites involved in coenzyme A synthesis were roughly equal to the control (Figure 8B and Supplementary Dataset S1).

Important cofactors such as ADP, ATP, and NADP^+^ were also significantly depleted (Figures 7A, 8A,C). Notably, pathway analysis revealed that changes in PPP and pantothenate and CoA biosynthesis made the greatest contribution to compensate for the fitness cost caused by overexpression of *mcr-1* (Figures 6–8).

## Discussion

The emergence of *mcr* genes has heightened concerns surrounding polymyxin resistance due to the potential for horizontal transfer (Andersson et al., 2020). It is now clear that *mcr-1* encodes a phosphoethanolamine transferase, an inner membrane protein which attaches phosphoethanolamine to the lipid A, reducing susceptibility to polymyxins (Liu et al., 2017). However, the expression of *mcr-1* also imposes a biological fitness burden on bacteria (Yang et al., 2017; Ma et al., 2018; Tietgen et al., 2018). Although many studies have examined the epidemiology of *mcr-1*, little is known about the downstream metabolic changes that occur in response to its expression. In this study we examined the impact of different levels of expression of *mcr-1* on bacterial growth at 8 and 24 h and investigated the dynamics of adaptive metabolic responses to its over-expression.

Similar to previous studies (Denervaud Tendon et al., 2017; Yang et al., 2017; Zhang et al., 2019), we found that the expression of *mcr-1* was orchestrated and associated with an initial fitness cost (reduced growth at 8 h). However, to the best of our knowledge our study is the first to examine in any bacterial species the impact of the over-expression of the *mcr-1* on bacterial metabolism. It has been shown that MCR-1 contains a periplasmic *C*-terminal domain and an *N*-terminal transmembrane domain, anchoring the protein within the inner membrane (Wanty et al., 2013; Ma et al., 2016). The MIC of colistin increased 4-fold to 2 mg/L in the presence of 0.2% L-arabinose while dropped to 1 mg/L in the presence of 2% L-arabinose. It is possible that the more over-expression of mcr-1 will form non-functional MCR-1 protein and then reduce the MIC value and the transmembrane domain of MCR-1 triggers the destruction of the membrane (Eckert and Beck, 1989; Coyne et al., 2011). Over-expression of membrane protein affects membrane integrity and consequently cell viability (Wagner et al., 2006), and saturation of the translocon during membrane protein over-expression may lead to the accumulation of cytoplasmic aggregates and broad perturbations in the proteome (Gubellini et al., 2011). Furthermore, the observed fitness cost at 8 h associated with over-expression of *mcr-1* was associated with changes in cell morphology (Figures 2D,H), leading to alterations in cell permeability and leakage of intercellular cytoplasm (Yang et al., 2017; Li B. et al., 2019). Overall, our observations suggest that the presence of the MCR-1 protein in the bacterial membrane plays the dominant role in the initial reduction in cell growth.

While the over-expression of *mcr-1* resulted in reductions in bacterial growth over the first 8 h as described above, in each case growth had returned to control levels by 24 h (Figure 1B). This observation correlated well with our RT-PCR data that showed the expression of *mcr-1* was high at 8 h but had markedly dropped at 24 h (Figures 1C,D). This suggests that over-expression of *mcr-1* has a detrimental impact on bacterial viability and also that to relieve the associated burden bacteria defend themselves by limiting the expression of *mcr-1*, eventually returning to normal levels of growth (Bontron et al., 2016; Denervaud Tendon et al., 2017). Specially, at 8 h, the expression of *mcr-1* was greatest with 0.02% L-arabinose (Figure 1B), despite greater reductions in growth (∼1.5 log_10_ CFU/mL) with the higher L-arabinose concentrations (0.2 and 2%; Figure 1D). It is possible that maximizing the production of MCR-1 is accompanied by compensatory responses that enable *mcr-1* to be expressed at high levels with relatively low adverse effects (Schlegel et al., 2012; Yang et al., 2017). By 24 h, all bacteria had entered the stationary phase, but no expression of *mcr-1* was detected in these bacterial with or without L-arabinose induction (Figure 1D). This may be due to the lack of essential nutrients and the accumulation of metabolic products in stationary phase, which results many metabolism-linked genes including *mcr-1* switched off (Nystrom, 2004; Jaishankar and Srivastava, 2017).

Our study focused on the impact of differential expression of *mcr-1* on bacterial growth at different times to investigate the dynamics of adaptive metabolic responses to the over-expression of *mcr-1*. Our metabolomics results demonstrated that the over-expression of *mcr-1* led to perturbations in the metabolism of lipids, amino acids, carbohydrates and nucleotides. Major changes were observed in the metabolism of lipids, including fatty acid biosynthesis and elongation, glycerophospholipid (GPL) biosynthesis and degradation (Figures 4, 5). These metabolic changes are primarily associated with cell envelope biosynthesis (Maifiah et al., 2017). This suggests that the over-expression of *mcr-1* interferes with the synthetic process of the cell envelope leading to membrane damage and reduced viability (Wagner et al., 2006, 2007). The widely shared metabolomic adaptive changes affecting membrane lipids in MCR-1, especially glycerophospholipid (GPL) metabolism, further demonstrate that the anchoring of the protein within the inner membrane interferes with membrane integrity and imposes a fitness cost. This is consistent with our results (Figures 2, 4, 5) and previous reports that over-expression of *mcr-1* alters bacterial morphology (Yang et al., 2017; Li H. et al., 2019).

At 8 h, the over-expression of the proteins MCR-1 on membrane lipids resulted in a large accumulation of the short and medium-chain fatty acids which are important substrates for glycerophospholipid synthesis. In contrast, there was a decline in glycerophospholipid (GPL) metabolism which included decreased levels of phosphatidylethanolamine (PE), phophatidylglycerol (PG), and cardiolipin, the major membrane phospholipids in *E. coli* (Nikaido and Vaara, 1985; Nikaido, 2003; Tan et al., 2012). The membrane lipid composition is not fixed and a great diversity of amphiphilic lipids are produced by bacteria in order to adapt unfavorable conditions (Sohlenkamp and Geiger, 2016). Therefore, the metabolic changes observed in lipid biosynthesis which results in changes in the lipid composition of the membrane primarily—may assist in overcoming the potential fitness costs due to the over-expression of *mcr-1* (Figures 4, 5). Furthermore, at 24 h bacteria carrying *mcr-1* displayed equivalent growth rates to that of the control group (*mcr-1* negative strain) despite continued changes in their lipid metabolism. Combined with our TEM results that indicated intact membranes at 24 h (Supplementary Figure S1), this shows the bacterial cells were able to synthesize major membrane phospholipids and form intact membranes (Henderson et al., 2016; Sohlenkamp and Geiger, 2016). These data indicate that bacteria with a lower level of *mcr-1* expression are more likely to regain membrane integrity at 24 h (Supplementary Figure S1).

Finally, in addition to an increase in lipid metabolism, the over-expression of *mcr-1* substantially reduced the levels of purines and pyrimidines, amino sugars, carbohydrates, nucleotides and secondary metabolites (Figure 4). It is likely that the changes observed in lipid metabolism affected other metabolic pathways (Jozefczuk et al., 2010). Interestingly, enrichment and pathway analysis revealed the over-expression of *mcr-1* had the most effect on pentose phosphate pathway and pantothenate and CoA biosynthesis at 8 h (Figures 6–8) when over-expression of *mcr-1* has a detrimental impact on bacterial viability (Figure 1B). Moreover, the perturbations in pantothenate and CoA biosynthesis, together with a reduction in energy metabolism (ATP, ADP, NADP^+^), suggests an association between a reduction in pantothenate and CoA biosynthesis and energy with the expression of *mcr-1*. It has been shown that pantothenate and CoA play crucial roles in membrane lipid synthesis and cellular metabolism in Gram-negative bacteria (Begley et al., 2001). In particular, CoA is essential for the biosynthesis of lipid A through its involvement in short- and long-chain fatty acid biosynthesis (Leonardi et al., 2005; Needham and Trent, 2013). It is currently unclear which reactions or factors are affected by *mcr-1* and thereby induce the down regulation of pentose phosphate pathway, pantothenate and CoA biosynthesis and energy. Further experiments to elucidate the mechanisms by which this occurs are warranted.

## Conclusion

This study demonstrates that the expression of *mcr-1* causes global metabolic perturbations in bacteria that imposes a fitness cost. In order to recover from the physiological burden imposed, bacteria limit the over-expression of *mcr-1*. The metabolic responses to over-expression in *E. coli* mainly involve disturbances in cell envelope biogenesis and increased lipid metabolism. Notably, the over-expression of *mcr-1* is closely linked to impairment of pentose phosphate pathway, pantothenate and CoA biosynthesis and energy. The multi-level metabolomic changes observed increase understanding of the physiological adaptations that bacteria adopt to cope with *mcr-1* expression and therefore coping with colistin resistance. This may lead to the identification of therapeutic targets for future treatment.

## Data Availability Statement

All datasets generated for this study are included in the article/Supplementary Material.

## Author Contributions

J-HL and JLi designed the study. Y-YL, Q-LZ, JLu, X-QZ, HW, MA, and TL carried out the experiments. Y-YL, YZ, SN, and M-LH analyzed the data. Y-YL, PB, J-HL, and JLi drafted and revised the manuscript. All authors contributed to the article and approved the submitted version.

## Conflict of Interest

The authors declare that the research was conducted in the absence of any commercial or financial relationships that could be construed as a potential conflict of interest.

## Footnotes

1 http://mzmatch.sourceforge.net/ideom.php

## Supplementary Material

Supplementary Figure S1 TEM micrographs of *E. coli* at 24 h with or without induction by 0.2% L-arabinose.

Supplementary Table S1 Plasmids and strains used in this study.

Supplementary Table S2 Primers used in this study.

Supplementary Table S3 The median relative standard deviation (RSD) for all metabolites.

Supplementary Dataset S1 All of 1,101 putatively identified unique metabolites.
