# Supplementary material for: Metabolic Perturbations Caused by the Over-Expression of mcr-1 in Escherichia coli
Source: Front Microbiol. 2020 Oct 9;11:588658. doi: 10.3389/fmicb.2020.588658 (PMC7581681; doi:10.3389/fmicb.2020.588658)
Supplement: Supplementary Figure S1 — TEM micrographs of E. coli at 24 h with or without induction by 0.2% L-arabinose. [file Image_1.pdf]

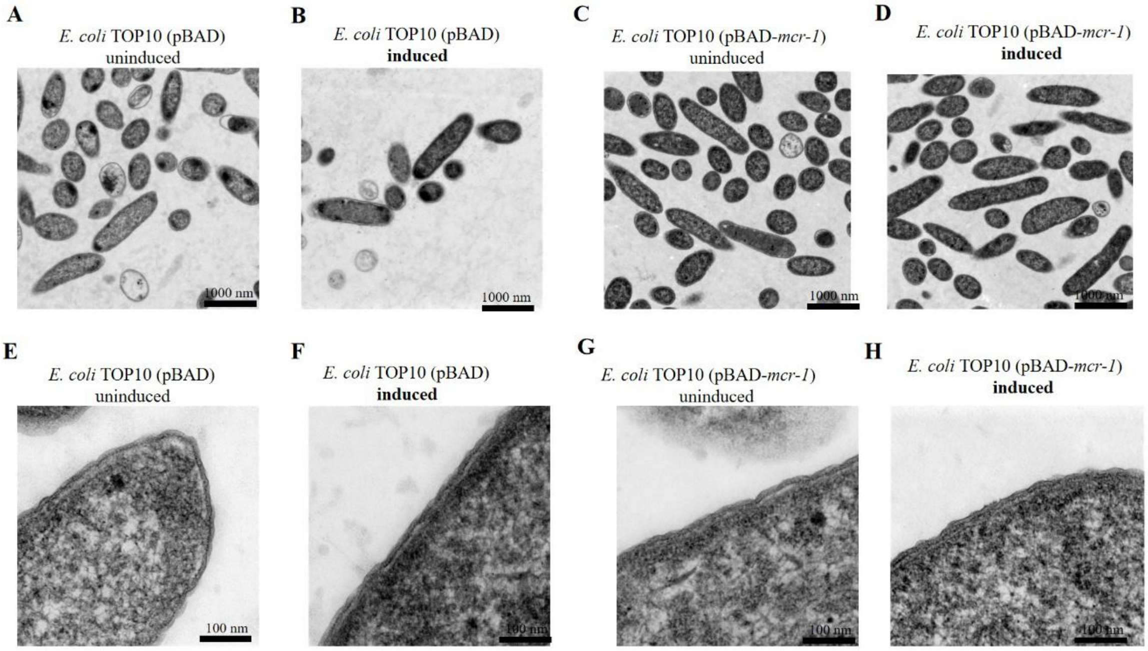

Figure S1. TEM micrographs of *E. coli* at 24 h with or without induction by 0.2% L-arabinose. (A) and (B) show *E. coli* TOP10 + pBAD (the vector control) and (C) and (D) *E. coli* TOP10 + pBAD *mcr-1*. (E) to (H) show partially magnified images of figures (A) to (D), respectively. Most cells had an intact membrane and the electron density of cytoplasmic region is homogeneous.
