# Supplementary material for: Metabolic Perturbations Caused by the Over-Expression of mcr-1 in Escherichia coli
Source: Front Microbiol. 2020 Oct 9;11:588658. doi: 10.3389/fmicb.2020.588658 (PMC7581681; doi:10.3389/fmicb.2020.588658)
Supplement: Supplementary Table S1 — Plasmids and strains used in this study. [file Table_1.docx]

**Table S1.** Plasmids and strains used in this study

| **Plasmid/Strain Relevant characteristics** | | **Origin** |
| --- | --- | --- |
| pBAD Plasmids, an arabinose inducible expression vector, Amp^R^ | | Lab stock |
| pBAD-*mcr-1* Plasmids, the pBAD carrying *mcr-1* | | This work |
| *E. coli* TOP10 Strains, a wild-type *E. coli* used as a hosting strain | | Lab stock |
| **Strains** | **L-Arabinose (w/v) Induction** | **MIC (mg/L)** |
| *E. coli* TOP10 + pBAD | 0% | 0.5  0.5  0.5  0.5 |
|  | 0.02% |  |
|  | 0.2% |  |
|  | 2% |  |
| *E. coli* TOP10 + pBAD-*mcr-1* | 0% | 0.5 |
|  | 0.02% | 0.5 |
|  | 0.2% | 2 |
|  | 2% | 1 |
