# Supplementary material for: Metabolic Perturbations Caused by the Over-Expression of mcr-1 in Escherichia coli
Source: Front Microbiol. 2020 Oct 9;11:588658. doi: 10.3389/fmicb.2020.588658 (PMC7581681; doi:10.3389/fmicb.2020.588658)
Supplement: Supplementary Table S2 — Primers used in this study. [file Table_2.docx]

Table S2. Primers used in this study

| **Primer** | **Sequencing (5'-3')** | **Note** | **Reference** |
| --- | --- | --- | --- |
| *mcr-1*-NS-F | GGAATTCCATATGATGCAGCATACTTCTGTGT | Restrict enzyme, NdeI | This study |
| *mcr-1*-NS-R | ACGCGTCGACTCAGCGGATGAATGCGGTGC | Restrict enzyme, SalI |  |
| pBAD-NS-F | ATGCCATAGCATTTTTATCC | - |  |
| pBAD-NS-R | GATTTAATCTGTATCAGG | - |  |
| *mcr-1*-Fw_6 | GCAGCATACTTCTGTGTGGTAC | qPCR | This study |
| *mcr-1*-Rv_134 | ACAAAGCCGAGATTGTCCG |  |  |
| *rpoB*-F | TCCTTTCTATCCAGCTTGACTCGT |  | (Yang et al., 2017) |
| *rpoB*-R | CGCAGTTTAACGCGCAGCGG |  |  |

References

Yang, Q., Li, M., Spiller, O. B., Andrey, D. O., Hinchliffe, P., Li, H., et al. (2017). Balancing *mcr-1* expression and bacterial survival is a delicate equilibrium between essential cellular defence mechanisms. *Nat. Commun.* 8:2054.
