# Supplementary material for: Metabolic Perturbations Caused by the Over-Expression of mcr-1 in Escherichia coli
Source: Front Microbiol. 2020 Oct 9;11:588658. doi: 10.3389/fmicb.2020.588658 (PMC7581681; doi:10.3389/fmicb.2020.588658)
Supplement: Supplementary Table S3 — The median relative standard deviation (RSD) for all metabolites. [file Table_3.docx]

Table S3. The median relative standard deviation (RSD) for all metabolites

| **8 h** |  |
| --- | --- |
| pBAD, NO induction | 28.2% |
| pBAD, Arabinose induction | 32.7% |
| pBAD-*mcr-1*, NO induction | 20.0% |
| pBAD-*mcr-1*, Arabinose induction | 24.5% |
| **24 h** |  |
| pBAD, NO induction | 36.7% |
| pBAD, Arabinose induction | 24.2% |
| pBAD-*mcr-1*, NO induction | 22.9% |
| pBAD-*mcr-1*, Arabinose induction | 24.4% |
| **PBQCs** | 16.5% |

Data precision of individual samples represented as the median relative standard deviation (RSD) for all metabolites based on all replicates (*n* = 5) of each group (*n* = 6 for technical replicates of PBQCs).
